# Adaptive Response of Thermophiles to Redox Stress and Their Role in the Process of dye Degradation From Textile Industry Wastewater

**DOI:** 10.3389/fphys.2022.908370

**Published:** 2022-06-20

**Authors:** Tadele Assefa Aragaw, Fekadu Mazengiaw Bogale, Amare Gessesse

**Affiliations:** ^1^ Faculty of Chemical and Food Engineering, Bahir Dar Institute of Technology, Bahir Dar University, Bahir Dar, Ethiopia; ^2^ Institute of Biotechnology, Addis Ababa University, Addis Ababa, Ethiopia; ^3^ Department of Biological Sciences and Biotechnology, Botswana International University of Science and Technology, Palapye, Botswana

**Keywords:** dyes, redox response, thermophiles, stress, antioxidant, enzymes, regulators, reactive oxygen species (ROSs)

## Abstract

Release of dye-containing textile wastewater into the environment causes severe pollution with serious consequences on aquatic life. Bioremediation of dyes using thermophilic microorganisms has recently attracted attention over conventional treatment techniques. Thermophiles have the natural ability to survive under extreme environmental conditions, including high dye concentration, because they possess stress response adaptation and regulation mechanisms. Therefore, dye detoxification by thermophiles could offer enormous opportunities for bioremediation at elevated temperatures. In addition, the processes of degradation generate reactive oxygen species (ROS) and subject cells to oxidative stress. However, thermophiles exhibit better adaptation to resist the effects of oxidative stress. Some of the major adaptation mechanisms of thermophiles include macromolecule repair system; enzymes such as superoxide dismutase, catalase, and glutathione peroxidase; and non-enzymatic antioxidants like extracellular polymeric substance (EPSs), polyhydroxyalkanoates (PHAs), etc. In addition, different bacteria also possess enzymes that are directly involved in dye degradation such as azoreductase, laccase, and peroxidase. Therefore, through these processes, dyes are first degraded into smaller intermediate products finally releasing products that are non-toxic or of low toxicity. In this review, we discuss the sources of oxidative stress in thermophiles, the adaptive response of thermophiles to redox stress and their roles in dye removal, and the regulation and crosstalk between responses to oxidative stress.

## Graphical Abstract

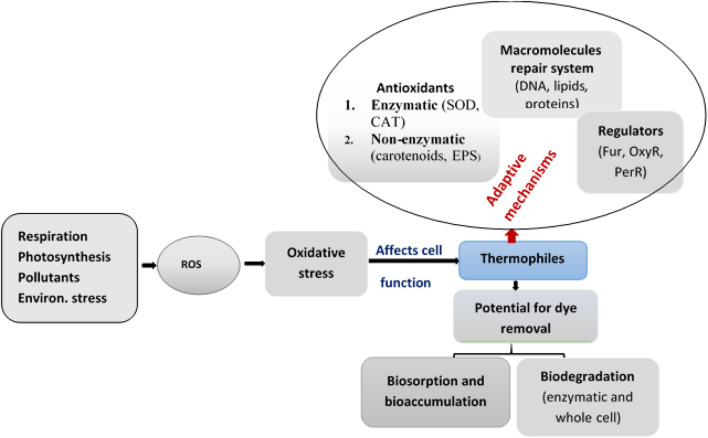

## 1 Introduction

To meet varied customer choices the textile industry uses different dyes in the manufacture of fabrics having a range of colors. Today different classes of dyes are in use by the textile industry that vary in chemical compositions and other properties (Berradi et al., 2019). In the dying process, out of the total dye added to the dying bath, the fabric takes only about 60% and the remaining 40% is released as waste (Berradi et al., 2019). As a result, the textile industry is one of the major sources of organic dyes posing serious challenges of environmental pollution (Mustafa et al., 2021).

The dyes used by the textile industry differ from one another based on their chemical structure, composition, and their other properties. Therefore, dyes can be classified based on their origin as natural and synthetic; based on applications as vat dyes, dispersive dyes, and azoic colors; or based on chemical structures as acridine, anthraquinone, nitroso, and azo dyes. Dyes can also be classified based on the nature of their ions, as cationic (basic dyes), anionic (direct, acid, and reactive dyes), and nonionic (disperse dyes) (Vikrant et al., 2018).

Release of untreated textile wastewater could pose multiple environmental challenges. First, the presence of a high concentration of dye alters the color of water and affect its esthetic quality. Second, the presence of dye in lakes and rivers interferes with light penetration affecting photosynthetic activities and threatening the normal biological function of such water bodies. Third, many of the dyes and their degradation products are toxic, and some are carcinogenic affecting human and animal health (Lellis et al., 2019). Therefore, dye-containing wastewater released by the textile industry needs to be treated using appropriate wastewater treatment processes. However, the presence of different classes of dyes that differ in their chemical properties pose challenges to developing efficient processes for the treatment of textile industry effluents.

To remove dyes from textile industry wastewater, different physicochemical methods have been used. However, most of these treatment techniques are expensive and not environmentally friendly (Aragaw and Bogale, 2021). On the other hand, biological treatment methods are cheap, efficient, and environmentally safe (Aragaw, 2021). Biological treatment processes rely on the use of microbial consortia for the degradation and removal of dyes (Pinheiro et al., 2022). Microorganisms that grow under different conditions of pH (from neutral to alkaline), temperature (ambient temperature to high temperature), or salinity (from low to high salt concentration) are known to degrade dyes. Some of the dyes and the intermediate products generated during the degradation process, such as aromatic amines, are known to cause oxidative stress to microorganisms involved in the degradation process (Giovanella et al., 2020) and could affect the efficiency of dye degradation (Liu et al., 2021). When challenged with oxidative stress microorganisms protect themselves by producing different enzymes, such as superoxide dismutase and catalase (Bedekar et al., 2014), NADH: quinone oxidoreductase (Gianolini et al., 2020), glyoxal oxidase (MtGLOx), and other extracellular oxidoreductases (Yan et al., 2021). Therefore, understanding the adaptive response mechanisms of microorganisms to oxidative stress could play an important role in the design of treatment processes (Chen G. et al., 2021).

Extremophiles survive and optimally grow in extreme environments (high or low temperature, extremes of pH, or high salinity) and thus evolve adaptive mechanisms of survival which other microorganisms lack (Giovanella et al., 2020). These adaptive mechanisms could also include resistance to oxidative stresses (Orellana et al., 2018; Jeong and Choi, 2020). In recent years there has been growing attention given to the application of thermophiles in different industrial processes. However, most research attention is focused on the use of thermophiles as sources of thermostable industrial enzymes. Thermophiles have also been shown to have interesting applications in the bioremediation of heavy metals and for the degradation of different organic molecules, including dyes (Nzila, 2018). Thermophiles are expected to exhibit a high capacity to adapt to oxidative stress, including oxidative stress arising during dye degradation (Baker et al., 2021). In this review the potential of thermophiles to bring about dye degradation and the adaptive mechanisms they employ to resist dye-induced oxidative stress is discussed.

## 2 Dye Types Used by the Textile Industry, Their Environmental Impact, and Biological Degradation

### 2.1 Dye Types Used by the Textile Industry

The textile industry uses natural or synthetic dyes for the dyeing of fabrics. Though natural dyes are considered environmentally friendly and possess other desirable properties (such as being safe to human skin and imparting a good appearance to the fabric), their bonding capability to textile fibers is relatively poor. In addition, the availability of raw materials used for the extraction of natural dyes varies depending on geographic location, weather conditions, and seasonal variation making their production highly unsustainable (Kumar Gupta, 2019). On the other hand, different kinds of synthetic dyes can be produced as required from intermediates widely available in the chemical industry and are widely used by the textile industry (Khan and Malik, 2014; Yaseen and Scholz, 2019).

The different classes of synthetic dyes used by the textile industry include azo dyes, anthraquinone, triarylmethane, sulfur, indigo, azine, oxazine, thiazine, xanthene, nitro, nitroso, methine, thiazole, indamine, indophenol, lactone, aminoketone, hydroxyl ketone, and phthalocyanine (Selvaraj et al., 2021). Based on their mode of application, synthetic dyes can also be grouped as direct, reactive, disperse basic, acidic, metal complex, mordant, sulfur, and vat type of dying (Table 1) (Singh et al., 2020; Verma et al., 2021). Based on the structure of chromophores, dyes can be classified as azo (monoazo, disazo, triazo, polyazo), anthraquinone, phthalocyanine, and triarylmethane (Table 1). For example, the synthetic azo dyes have a common azo linkage (–N = N–), called azo bonds (Gallo et al., 2021). In addition to differences in chemical structure and properties, these dyes also differ in their susceptibility to microbial degradation.

**TABLE 1:** Some common classifications of dyes based on their chemical composition or application. Adopted with modification from (Rauf and Salman Ashraf, 2012; Shah, 2019; Giovanella et al., 2020; Ihsanullah et al., 2020).

No	Dye type	Description	Possible chemical structures
1	Acid dyes	Have pH in the range of 3–7 that is applied under acidic conditions, they have a variety of structures and metal complex	Anthraquinone, xanthene, azo (including, nitroso, pre-metalized), nitro, and triphenylmethane
2	Sulfur dyes	have a highly complex structure, made mainly by thionization of several aromatic intermediates	Indeterminate structures
3	Basic dyes	have diffusion in hot water, they produce cation to attract a negative charge to dye	Hemicyanine, azo, cyanine, diazahemicyanine, azinediphenylmethane, xanthene, triarylmethane, acridine, anthraquinone, and oxazine
4	Disperse dyes	are insoluble in water and applied in the hydrophobic substrate	Azo, nitro, anthraquinone, benzodifuranone, and styryl
5	Direct dyes	are formed by several compounds such as chromophoric, stilbene, phthalocyanine, dioxazine, and other smaller chemicals	Phthalocyanine, azo, oxazine, and stilbene
6	Reactive dyes	are a group of dyes with specific functional groups that acts by a covalent bond with the substrate	Anthraquinone, formazan, phthalocyanine, azo, oxazine, and basic dye
7	Vat dyes	The dye can be made soluble in water dropping in sodium hydrogen sulfite, and applied to the fiber.	Indigoids and anthraquinone Sulphur

### 2.2 Effect of Textile Dyes on the Environment and dye Degradation by Thermophiles

The release of textile dye-containing wastewater into water bodies causes severe environmental pollution. First, as organic molecules, the presence of dyes increases the chemical oxygen demand (COD), and biological oxygen demand (BOD) of aquatic ecosystems and affects all life forms (Gallo et al., 2021). Second, the presence of dyes decreases light transmission and affects photosynthetic activity in aquatic ecosystems. Third, many dyes and their degradation products are toxic and some of them are highly persistent in the environment (Navas et al., 2020).

In some cases, intermediates generated in the process of dye degradation, such as aromatic amines, could be more toxic than the starting dye molecules (Balapure et al., 2015) some of them causing oxidative stress. Such molecules are usually formed in oxygen-limited environments (Bhatia et al., 2017). But in the treatment processes involving sequential anaerobic-aerobic systems, the intermediate products could be completely oxidized and prevent the accumulation of toxic intermediates (Shoukat et al., 2019).

Most dyes are highly stable upon exposure to light and many are resistant to microbial degradation which results in their accumulation in the environment (Puvaneswari et al., 2006) posing serious ecological risks (Shah, 2019). Compared to textile dyes with hydroxyl or amino functional groups, azo dyes containing methoxy, methyl, sulfo, or nitro groups are considered more toxic and resistant to microbial degradation (Pinheiro et al., 2022). This is because the azo dye having a functional group with higher electronic density does not favor a second electron transfer forming the dianion resulting in poor or no decolorization (Pearce et al., 2003). Within the azo dye groups, sulfonated azo dyes are more resistant to microbial degradation than carboxylated azo dyes because of their physicochemical characteristics (Saratale et al., 2011).

In general, compared to dyes having a high molecular weight and complex structure, those with low molecular weight and simple structure are easier to decolorize probably because of minimal steric effect (Varjani et al., 2020). In addition to the molecular structure, the presence of high dye concentration affects microbial degradation processes. As the level of dye increases its toxicity also increases affecting microbial growth and thus reducing the available biomass to catalyze the degradation process. (Khan et al., 2013).

Microbial breakdown of synthetic dyes plays an important role in preventing pollution and the degradation process involves the action of different enzymes. However, the enzymes that play a critical role in the degradation process remain relatively unexplored. In recent years thermophilic microorganisms attracted increased attention as sources of important thermostable enzymes, including those involved in dye degradation (Mehta et al., 2016). Many thermophilic microbial species convert toxic azo dyes and/or their intermediates into low toxicity metabolites at elevated temperatures. This indicates that thermophiles have metabolic pathways for the degradation and detoxification of textile dyes (Chen G. et al., 2021).

Although several thermophilic bacteria were isolated from different thermal environments and evaluated for a range of biotechnological applications (Mehta et al., 2016), relatively little effort has been made to study thermophiles involved in dye degradation. However, lab-scale studies of thermophiles isolated from hot springs showed their potential for the removal of various dyes (Mohammad et al., 2017). In another report, immobilized cells of *Geobacillus stearothermophilus* ATCC 10149, a thermophilic bacterial strain, were tested for the degradation of the dye Remazol Brilliant Blue R (RBBR) which resulted in 90% removal (Gianolini et al., 2020). This strain was shown to produce a thermostable laccase that is involved in the decolorization of RBBR and other closely related dyes like Methyl Orange, Malachite Green, and Indigo Carmine. Similarly, another thermophilic bacterial strain, Anoxybacillus sp. PDR2, was shown to effectively degrade Direct Black G (DBG), a dye known to be highly toxic to most organisms (Chen G. et al., 2021).

The mechanism used by thermophilic microbial strains for dye removal involves binding of the dye molecules to the microbial cell through specific functional groups on the cell surface (biosorption), accumulation inside the cell (bioaccumulation), and direct breakdown of the dye molecule which serves as a substrate, or a combination of these mechanisms (Singh and Singh, 2017)*.*

### 2.3 Biosorption and Bioaccumulation of Dyes

Biosorption involves the removal of dyes through adsorption onto live or dead microbial biomass while bioaccumulation involves active uptake and intracellular accumulation of the dye molecule inside live cells (Mahmood et al., 2016). The presence of different functional groups in the cell wall of microorganisms, such as alcohol, aldehydes, ketones, carboxylic, ether, and phenolic groups, allow strong binding of dye molecules (Siddiqui et al., 2018). Biological materials including chitin, peat, chitosan, yeast, and fungi biomass are frequently used in the sorption of dye from solution through the mechanism of chelation and complexion (Almeida and Corso, 2019). Extracellular polymeric substances (EPSs) that mainly consist of biomolecules such as proteins, polysaccharides, and nucleic acids found in different bacteria functional groups (such as carboxyl and hydroxyl groups) that are negatively charged are known to be involved in the chelation of metal cations and prevent the cell from toxic metals (Zeng et al., 2020)*.* In addition, EPSs also bind to metal bonded synthetic dyes and facilitate its biosorption of negatively charged dyes (Wu et al., 2021).

The thermophilic cyanobacteria *Phormidium sp*, which grows at pH 8.5 and 45°C was reported to bioaccumulate the reactive dyes Black B and Remazol Blue when exposed to initial dye concentrations of 11.8 to up to 84.5 mg/L (Sadettin and Dönmez, 2007). Similar observations of reactive dye bioaccumulation have also been reported for the thermophilic cyanobacterial strain *Synechococcus sp* when exposed to an initial dye concentration of up 25 mg/L (Sadettin and Dönmez, 2006).

### 2.4 Enzyme Mediated dye Degradation

Dye biodegradation involves a step-by-step breakdown of the molecule in a cascade of reactions catalyzed by specific enzymes (Karthik et al., 2016). These processes are normally carried out under anaerobic, aerobic, or *via* sequential anaerobic-aerobic conditions resulting in the partial or complete oxidation of the dye molecule (Singh and Singh, 2017). Different enzymes are involved in dye degradation out of which laccase is one of the most important. Apart from their role in degradation inside cells, because of their high thermostability, laccases from thermophiles could also offer interesting potential for enzymatic dye degradation.

**FIGURE 1:**
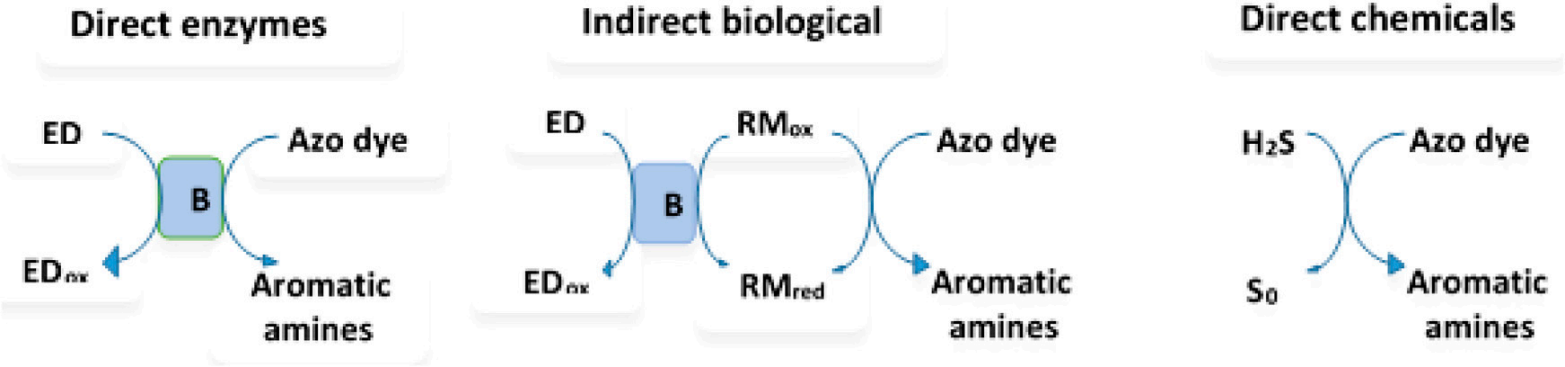
Different mechanisms of the azo dye reduction (B represents bacteria (enzyme system); ED = electron donor; RM = redox mediator). Modification from (Shah, 2019).

As shown in Figure 1, dye degradation could involve direct enzymatic, non-enzymatic biological degradation, or direct chemical degradation (Shah, 2019). During enzymatic oxidation of azo dyes, the electron withdrawal groups could make the compounds undergo oxidative catabolism.

**FIGURE 2:**
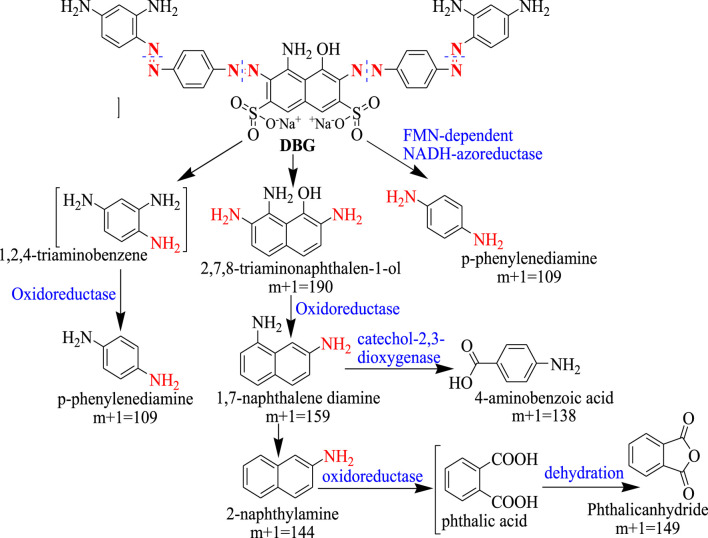
Enzymatic mechanism for the degradation of Direct Black G dye by *Anoxybacillus sp*. PDR2. Adopted with Re-drawn from (Chen et al., 2021).

Thermophiles that are capable of degrading lignin from lignocellulosic biomass have been shown to efficiently decolorize textile dyes. Lignin degrading thermophiles produce different enzymes involved in lignin degradation which include lignin peroxidase, manganese peroxidase, and laccase (Lai et al., 2017). These same enzymes have also been shown to be involved in dye degradation (Sharma and Vasanth, 2018). Lignin peroxidase catalyzes the oxidative cleavage of C-C and C-O-C bonds in a broad variety of organic compounds and, while doing so, generates free radicals (Guerriero et al., 2015; Janusz et al., 2017). For example lignin peroxidase was reported to be involved in the degradation of different dyes, such as Metanil yellow G (Guo et al., 2021), Congo red (Kishor et al., 2021), and Direct Black (Chen et al., 2018). In addition, different thermophilic microorganisms, such as *T. thermophilus, Anoxibacillus, Geobacillus, Thermosediminibacter,* were reported to produce peroxidases (DyP), laccases, and azoreductases all of them involved in dye decolorization (Gallo et al., 2021). Figure 2 shows the mechanism of degradation of Direct Black G dye by *Anoxybacillus sp*. PDR2 involving the action of different enzymes.

The enzymes involved in dye degradation by *Anoxybacillus sp*. PDR2 includes an NADH-azoreductase, an acyl-CoA dehydrogenase (ACADs), NADH-FMN oxidoreductases, and NADH: ubiquinone oxidoreductases (Chen et al., 2021). The first step of the reaction involves cleavage of the azo groups of the dye molecule into 2, 7, 8-triaminonaphthalen-1-ol (m+1 = 190), p-phenylenediamine (m+1 = 109), and 1, 2, 4-triamino-benzene is catalyzed by the FMN-dependent NADH-azoreductaze, a key enzyme in the degradation of the azo dye molecule, DGB. For subsequent degradation of the intermediates generated in the first reaction, two different pathways were confirmed. In the first pathway, a quinone oxidoreductase (type IV) catalyzes the oxidation of 1, 2, 4-triaminobenzene to p-phenylenediamine. In the second pathway, a part of amino and hydroxyl groups was removed from 2, 7, 8-triaminonaphthalen-1-ol by the action of oxidoreductases leading to the formation of 1, 7-naphthalene diamine (m+1 = 159). Further oxidation is catalyzed by catechol 2,3-dioxygenase generating 4-Aminobenzoic acid (m+1 = 138) or converted to phthalic acid and subsequently to phthalic anhydride (m+1 = 149) by the action of an oxidoreductase (Figure 2).

## 3 Oxidative Stress in Thermophiles

Oxidative stress (OS) is a major challenge encountered by microorganisms in many environments. It results from the imbalance between pro-oxidative and antioxidative species that are generated from endogenous or exogenous sources (Sen and Imlay, 2021)*.* Prooxidants that include reactive oxygen species (ROS) are produced mainly produced during aerobic respiration (Khaleque et al., 2020; Farías et al., 2021)*.* The ROS causes oxidative damage to cellular components by attacking nucleic acids, cell membranes, and proteins, which causes mutation, protein denaturation, enzyme inactivation, or lipid peroxidation and disrupts intracellular homeostasis (Stewart et al., 2012; Zhang et al., 2012; Ren et al., 2020)*.* The three main ROSs are superoxide (O_2_^−^), hydrogen peroxide (H_2_O_2_), and, hydroxyl radical (OH) which are produced either during microbial metabolic processes or following exposure to physical and chemical agents such as ionizing radiation, desiccation, ultraviolet radiation, and mitomycin (Gao et al., 2020). Increase in OH, H_2_O_2_, and O_2_^−^ concentration affects the normal functioning of the biological system, such as a reduction in ATP level. For example, in microorganisms ROSs cause cell deformation, disruption of the cell membrane integrity, and partial loss of the cytoplasm (Zhang H. et al., 2021)*.*

Under different growth conditions, thermophilic microorganisms are exposed to oxidative stress due to the accumulation of ROSs causing direct or indirect damage to different macromolecules, including metalloproteins and affecting their ability to maintain redox homeostasis (Farías et al., 2021)*.* Moreover, thermophilic bacterial cells could expose to various environmental stressors during the processes of dye degradation, including fluctuations in pH value, elevated pressure, and partial pressure of oxygen, and this could influence the oxidative stress responses (Obruca et al., 2021). For example, in *Pseudomonas putida,* high oxygen partial pressure influences the oxidative stress response where genes for various peroxidases and glutathione-related proteins are up-regulated (Farías et al., 2021)*.*

### 3.1 Sources of Oxidative Stress

Microorganisms can be exposed to different stresses that include oxidative stress, heat stress, cold stress, radiation, pH, nutritional, and stress due to exposure to pollutants (Ranawat and Rawat, 2017). Of these, oxidative stress caused by ROS generated in the process of aerobic respiration is the major stress microbial cells face. During respiration, a single electron is added to molecular oxygen resulting in the formation of the radical, superoxide ion. To protect themselves from the effect of the superoxide radical all aerobic cells have the enzyme superoxide dismutase that converts the superoxide ion into hydrogen peroxide (H_2_O_2_) (Latifi et al., 2009; Bueno et al., 2012). However, H_2_O_2_ chemically reacts with ferrous iron in what is known as the Fenton reaction and generates hydroxyl radical (•OH), a more reactive radical than superoxide ion. These two ROS generated inside the cell during aerobic respiration cause damage to macromolecules (such as DNA, proteins, and lipids) (Cabiscol et al., 2000). Free radicals can also be produced upon inadvertent autoxidation of flavoproteins (Farías et al., 2021; Fasnacht and Polacek, 2021) and during the enzymatic reduction of different aromatic compounds, including dyes.

**FIGURE 3:**
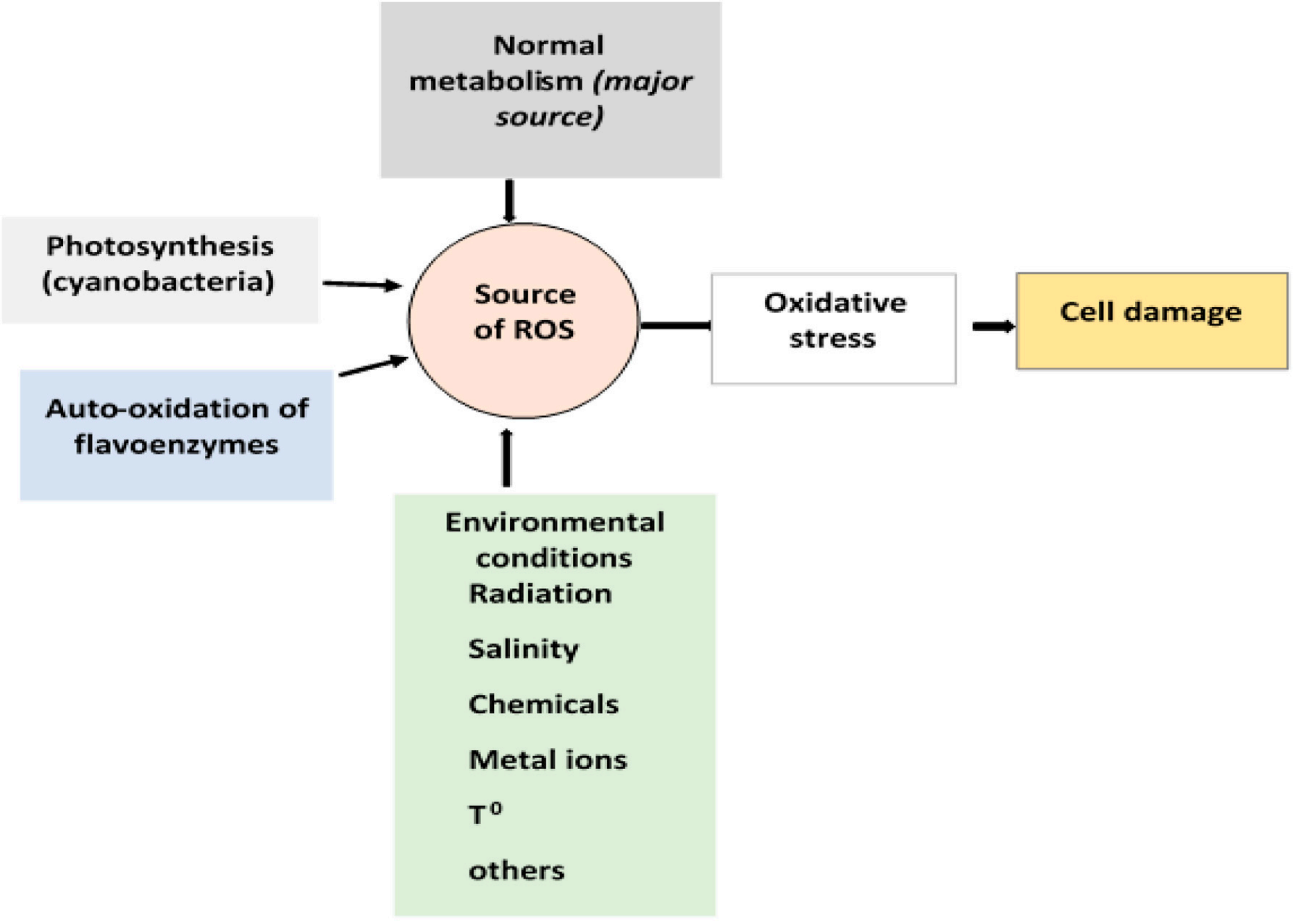
Sources of oxidative stress and its effect on cells.

Besides normal metabolic sources, environmental factors such as high oxygen pressure, lack of water, temperature, high metal ion concentrations, radiation, salinity, and other chemicals contribute to the generation of ROS (Figure 3) (Khaleque et al., 2020)*.* Many pollutants including dyes are redox-active, which, after uptake by microorganisms, cause univalent reduction of molecular oxygen, leading to the generation of superoxide radicals, H_2_O_2,_ and hydroxyl radical (•OH) (Zhang et al., 2012).

### 3.2 Adaptive Response to Oxidative Stress

Defense to oxidative stress (OS) by different groups of microorganisms involves both enzymatic and nonenzymatic mechanisms. These include an effective DNA repair system, prevention of the formation of endogenous ROS, activation of the antioxidant defense system, selective protection of the oxidative damage of certain proteins, and removal and degradation of damaged macromolecules (Mirończuk-Chodakowska et al., 2018; Gao et al., 2020). Energy dissipation and UV-sunscreens (for cyanobacteria) are also employed as preventative mechanisms (Latifi et al., 2009; Burton and Jauniaux, 2011). Production of reducing enzymes, ROS-detoxifying enzymes, antioxidant systems, and protein and DNA repair enzymes are involved as part of the adaptation mechanisms.

Although excessive oxidative stress leads to an undesirable effect on microbial cells, appropriate or balanced oxidative stress contributes to the activation of the antioxidant defense system (Sies, 2020; Zhang et al., 2021). The antioxidant defense system includes endogenous antioxidants both enzymatic and non-enzymatic such as superoxide dismutase (SOD), glutathione peroxidase (GPx), catalase (CAT), and glutathione (GSH), etc., and exogenous antioxidants such as vitamin E, vitamin C, polyphenols, and carotenoids (Bouayed and Bohn, 2010; Roehrs et al., 2011). Thus, antioxidative mechanisms determine the adaptation and abundance of microorganisms in the environment (Farías et al., 2021). This allows the DNA repair system and metabolic system to function normally and protect the cells against oxidative damage of the proteome (Gao et al., 2020).

The adaptive response mechanism against oxidative stress for microbial strains, such as *Streptococcus salivarius ssp thermophilus,* was evaluated (Riane et al., 2019) and was shown to contribute to the survival of the organism in natural and man-made environments (Farías et al., 2021). The presence of heat-stable antioxidant proteins was also shown to play a key role in the adaptation of thermophilic organisms to OS by eliminating excess free radicals (Graham et al., 2006)*.* Ianutsevich et al. (2020) reported the role of trehalose, a sugar-containing glucose molecule, functions as an antioxidant in the adaptation of the thermophilic fungus *Rhizomucor miehei* (Ianutsevich et al., 2020).

**FIGURE 4:**
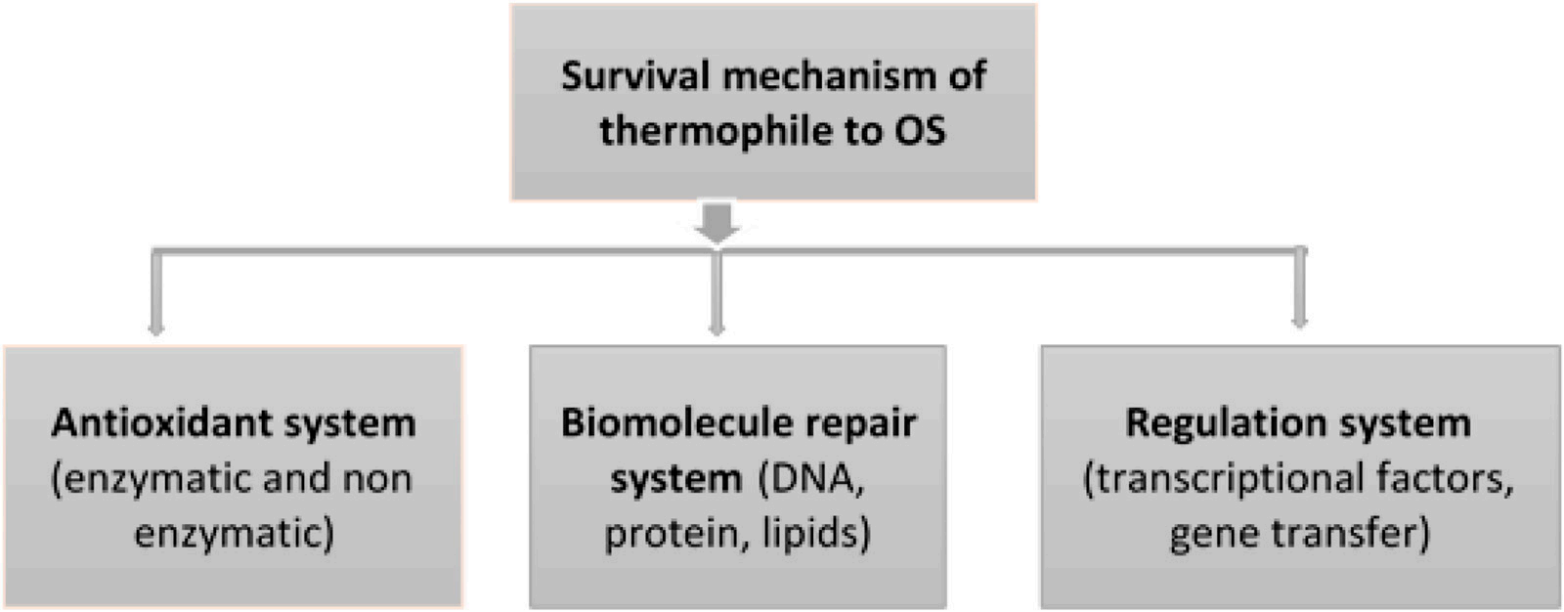
Response mechanisms of thermophiles and their regulation system to adapt oxidative stress.

As shown in Figure 4, the survival mechanism of thermophiles to oxidative stress involves an antioxidant system to scavenge ROSs, an integrated macromolecular repair system, and the regulation of different responses activated in the adaptive mechanisms (Latifi et al., 2009).

#### 3.2.1 Enzymatic Antioxidants and Enzymes Involved in dye Degradation

Microorganisms have evolved various enzymatic defense mechanisms to deal with the challenges of ROS. Superoxide dismutases (SODs), catalase (CAT), glutathione (GSH), glutathione peroxidase (GPx) alkyl hydroperoxide reductase (Ahp), peroxiredoxin (Prx), and others are common antioxidant enzymes involved in the scavenging of ROS (Akkoyun et al., 2020; Pedone et al., 2020). These enzymes are involved in redox homeostasis and are the core of the antioxidant system (Medvedkova et al., 2009; Pedone et al., 2020). The antioxidant enzyme levels differ greatly between different bacterial groups, for example between Gram-negative and Gram-positive strains.

Superoxide dismutases (SODs) are well-known antioxidant enzymes that play critical roles in the cellular defenses of living organisms against harmful superoxide radicals during oxidative stress (Pedone et al., 2020; Shahi et al., 2021). The most common iso-enzymes of SODs include copper-zinc SOD (Cu, ZnSOD), iron SOD (FeSOD), nickel SOD (NiSOD), and manganese SOD (MnSOD) (Ranawat and Rawat, 2017; Shahi et al., 2021). Recently, several novel SOD genes from various prokaryotic and eukaryotic organisms have been identified and characterized (Shahi et al., 2021). For example, the thermophile isolates *Thermus thermophilus* (HB27) isolated from thermal environment show excellent stability and activity under high temperatures, and also it is halotolerant (Chen N. W. et al., 2021). Iron-containing superoxide dismutase (CaSOD) was determined from a novel thermophilic bacteria, *Cohnella sp.* A01, and the gene was cloned and expressed in *E. coli* BL21. The recombinant protein (rCaSOD) was active in the pH range of 6.0–10.0 and the temperatures range of 35–75°C. Moreover, the rCaSOD was stable in the presence of high concentrations contaminants, organic solvents, and metal ions (Shahi et al., 2021).

CATs are involved in the decomposition of H_2_O_2_ and contribute to the defense of bacterial cells against OS (Atalah et al., 2020). They also play an important role in cellular processes including metabolite production (Yuan et al., 2021). Both SOD and CAT enzymes were observed in the thermophilic bacterial species of *E. profundum*.

A novel ferriperoxin (Fpx) (Rubrerythrin (Rbr)-like protein) was identified from *Hydrogenobacter thermophilus*. The Fpx exhibited two biological reactions, NADPH oxidoreductase (FNR)-dependent peroxidase activity and reduced both hydrogen peroxide (H_2_O_2_) and organic hydroperoxide in the presence of NADPH and FNR as electron donors, which is indicative of oxygen inhibition (Sato et al., 2012). In another study, ferredoxin oxidoreductase (PFOR) was investigated in the *Thermoanaerobacter kivui* in the glycolysis process, which can be converted into acetyl CoA that enters the Krebs cycle when adequate oxygen is available (Katsyv et al., 2021). Examination of the oxidative stress-induced proteins of the thermophilic bacterium *Bacillus stearothermophilus* TLS33 through proteomics revealed the presence of four isoforms of peroxiredoxin (Prx I, Prx II, Prx III, and Prx IV) after exposure to oxidative stress and the level of expression the different isoforms was not affected by prolonged oxidative stress (Topanurak et al., 2005). In hyperthermophilic archaea, SOD, peroxiredoxins, catalase, thioredoxin, thioredoxin reductase, and protein disulfide oxidoreductase were reported to be involved in the scavenging of ROS (Pedone et al., 2020).

Table 2 summarizes dye removal efficiency by selected thermophilic microbial strains and their response to oxidative stress. The microbial oxidative stress enzymes (SOD and CAT) both play a role in protecting the cell from OS generated during the degradation of dye, Reactive Orange 16 (RO16) (Bedekar et al., 2014). These enzymes were shown to be thermostable and are to degrade the dye at elevated temperatures (Chen G. et al., 2021). Other enzymes involved in dye degradation are azoreductases which are responsible for the reductive cleavage of the azo bonds (Lellis et al., 2019; Krithika et al., 2021), tyrosinase, lignin peroxidase, NADH-DCIP reductase, riboflavin reductase, and laccases (Dawkar et al., 2010; Saratale et al., 2011). *Aspergillus flavus* (fungus) (Mawad et al., 2020). In addition to thermophilic bacteria, thermophilic fungi such as *Myceliophthora thermophyla* (Kadowaki et al., 2018) produce enzymes involved in the detoxification of dyes.

**TABLE 2:** Enzymes produced from thermophile species in response to oxidative stress in the degradation process of textile dyes.

Thermophile species	Key enzymes produced in response to OS	Removed dye	% removal	Reference
*Lysinibacillus sp*. RGS	oxidoreductive enzymes (Superoxide dismutase and catalase activity)	Reactive Orange 16	100	Bedekar et al. (2014)
*Anoxybacillus sp.* PDR2	NADH: quinone oxidoreductase	Direct Black G (DBG)		Chen G et al. (2021)
*Geobacillus stearothermophilus* ATCC 10149	Extracted extracellular laccase	Remazol Brilliant Blue R	90	Gianolini et al. (2020)
*Myceliophthora thermophyla* (fungus)	glyoxal oxidase (MtGLOx), an extracellular oxidoreductase	aldehydes and α-hydroxy carbonyl substrates		Kadowaki et al. (2018)
*Caldanaerobacter* dominated extreme-thermophilic consortium (EX-AO7)	enzymes in response to OS are not reported	Acid Orange 7	100	Yan et al. (2021)
extreme-thermophilic mixed culture [*Caldanaerobacter* (64.0%) and *Pseudomonas* (25.4%)]	enzymes in response to OS are not reported	Acid Orange 7	90	Zhang et al. (2019)
*Geobacillus thermoleovorans*	enzymes in response to OS are not reported	Methylene Blue and Acid Orange G	100	Ibrahim et al. (2021)
*G. thermoleovorans* KNG 112	enzymes in response to OS are not reported	Amaranth RI and fast red E	90	Rajashekarappa et al. (2022)
*Bacillus* dominant halo-thermophilic bacterial consortium (HT1)	enzymes in response to OS are not reported	metanil yellow G (MYG)	92.6	Guo et al. (2021)
*Thermus sp. 2.9—*thermophilic bacterial strain	enzymes in response to OS are not reported	Xylidine,	98	Navas et al. (2020)
*Anoxybacillus rupiensis* Ir3 (JQ912241)	enzymes in response to OS are not reported	methylene blue and acid orange G	100	Mahdi et al. (2020)
*Novibacillus thermophiles *SG-1	gene encoding riboflavin biosynthesis protein	azo dye (Orange I)		Tang et al. (2018)
*Synechococcus sp*. and *Phormidium sp.,*	bioaccumulation	Remazol Blue, Reactive Black B, Reactive Red RB		Sadettin and Dönmez, (2006)

#### 3.2.2 Nonenzymatic Antioxidant Mechanisms

In addition to ROS-scavenging enzymes, microorganisms also employ non-enzymatic antioxidants such as manganese complexes, thioredoxin, α-tocopherol, carotenoids, exogenous glutathione (GSH), mycothiol, etc., to counter the impacts of OS (Latifi et al., 2009; Wu et al., 2020; Irato and Santovito, 2021). For example, mycothiol (MSH) was identified from a thermotolerant strain of *Corynebacterium glutamicum* (Irato and Santovito, 2021).

Thioredoxin is a redox protein that detoxify H_2_O_2_ which is then converted to the reduced form via thioredoxin reductase (Burton and Jauniaux, 2011). In addition, thioredoxin also plays a key role in protein repair in many bacteria (Zeller and Klug, 2006; Serrano et al., 2007). Polyhydroxyalkanoates (PHAs), extracellular polymeric substances (EPS), and other extracellular materials do also play important roles in protecting cells from OS damage by ROS (Steele et al., 2014). Therefore, together with other protective mechanisms (such as enzymatic antioxidants, repair mechanisms, and regulators), non-enzymatic antioxidants protect microbial cells from damage caused by ROSs.

## 4 Regulation of the Responses to Oxidative Stresses

Oxidative stress is caused by a variety of factors stated in the previous section and is controlled by complicated regulatory systems (Milisav et al., 2012). Some examples of genetic level oxidative stress regulations include transcriptional regulators (OxyR, PerR, etc.) (Gao et al., 2020; Fasnacht and Polacek, 2021), Nrf2 (Wang et al., 2017; Zhang Y. et al., 2021), tRNA methyl-transferase (Jaroensuk et al., 2016), and genes encoding UvrABC system (Hagi et al., 2018). The role of 2’-O-ribose tRNA methylation in the cell’s response to oxidative stress was investigated in *Saccharomyces cerevisiae* (Endres et al., 2020). For example, using 2’-O-ribose deletion mutants for Trms 3, 7, 13, and 44, in acute and chronic exposure settings to oxidative stress were investigated. A global analysis of H_2_O_2_-induced tRNA modifications showed a complex profile of decreased, or undetectable, 2’-O-ribose modification in 2’-O-ribose Trm mutant strains, showing a link between this type of modification event and Trm status post-exposure. For the thermophilic bacteria, *Thermus thermophilus* HB8 stationary phase-dependent regulatory protein (SdrP) regulate the expression of numerous genes involved in redox control, nutrient and energy supply, and nucleic acid metabolism. The same gene also plays a central role in the nucleotide excision repair of damaged DNA (Agari et al., 2010).

During dye degradation by *Deinococcus radiodurans* the regulatory mechanisms involved in the dye degradation processes include transcriptional regulators (members of AsnC, TetR, DdrI, and GntR families), damage response proteins, and DNA repair proteins (RecA, RecN) (Gao et al., 2020)*.*

OxyR and SoxR regulators are activated during OS that experience conformation changes during oxidation in the presence of H_2_O_2_ and superoxide radicals (Chiang and Schellhorn, 2012; Kim et al., 2015). For the Gram-negative bacteria, *Burkholderia thailandensis,* OxyR was found to be a conserved OS regulator important for the survival of the organism under oxidative stress (Si et al., 2017). *Campylobacter jejuni* modifies the expression of genes involved in OS resistance mainly by its OS regulator and the peroxide resistance regulator. This bacteria lacks SoxRS and OxyR that are observed in other Gram-negative species (Kim et al., 2015). Nitric oxide (NO) also serve as a cytoprotective system in *Bacillus subtilis* protecting cells from damage by ROS either through the reduction of free cysteine (that enhances Fenton reaction) or directly by activating catalase (Gusarov and Nudler, 2005).

## 5 Crosstalk Between Oxidative and Other Stresses

In the process of degradation of dyes and other contaminants, in addition to oxidative stresses, cells can also be exposed to different other stressors. These include redox balance, level of iron, salinity, and nitrosative stresses. These stresses could synergize and influence the degradation of dyes by different groups of microorganisms. However, susceptibility and response to these stresses differ among the different groups, such as mesophiles vs thermophiles.

### 5.1 Redox Balance

Most biochemical pathways in cells involve redox reactions, which highlights the critical importance of redox balance in the maintenance of homeostasis. Cellular protection against oxidative and electrophile toxicities (chemoprevention) can be provided either by redox-active, short-living direct antioxidants or indirect antioxidants activity (Valentová, 2020).

In the process of textile dye degradation, compared to mesophiles, thermophiles better resist the impact of OS resulting in balancing the redox reaction during the degradation process (Watkin et al., 2009). This has a direct relation with the structuring of water (including loss of rotational degrees of freedom), becoming less negative for the larger metal ions (Sawle and Ghosh, 2011). Glutathione (GSH) and thioredoxin (Trx) systems that scavenge harmful intracellular ROS are capable of controlling redox reactions. Redox reactions often involve molecules with thiol or sulfhydryl (-SH) functional groups, including low-molecular-weight (LMW) thiols, cystine-derived thiols, and redoxins (Ye et al., 2021). Intra-or intermolecular disulfide reduction by Trx is one example of redox-based regulation. Trxs are small abundant proteins involved in several cellular functions. They share a greatly conserved active site (Cys-Gly-Pro-Cys) that is part of a redox regulatory system in which electrons are transported from NADPH to thioredoxin reductase and finally to thioredoxins (Latifi et al., 2009). The involvement of Trxs in the response of many organisms to oxidative stress has been reported in detail (Zeller and Klug, 2006; Serrano et al., 2007; Burton and Jauniaux, 2011; Lu and Holmgren, 2014). From a metabolic perspective, the NADPH pool and the balance between the NADH and NADPH content play a vital role in controlling the redox state of the cell (Pedone et al., 2020). Redox reactions are also interrelated with different modes of regulation, e.g., between redox modifications and phosphorylation/dephosphorylation of proteins (Sies, 2020).

### 5.2 Iron

Iron is one of the metal ions required by bacterial and fungal cells playing important role in such physiological processes as DNA replication, transcription, metabolism, and energy generation via respiration. However, the presence of excess iron could also have a negative influence on microbial cells involved in the dye degradation. Hence, thermophilic bacteria and fungi developed sophisticated strategies to resist the negative impacts of high iron concentrations (Mishra et al., 2022).

The control of iron homeostasis and responses to OS are interrelated (Cornelis et al., 2011) because iron is an essential nutrient (a cofactor for many enzymes) for the growth of microorganisms. Iron can generate ROSs through the Fenton reaction (Cárdenas et al., 2012; Sen and Imlay, 2021) where H_2_O_2_ reacts with Fe^2+^ (Equ 1) to form the highly reactive hydroxyl radical (.OH) which is a strong oxidizing agent among the ROS leading to damage of DNA, proteins, lipids, and carbohydrates (Khaleque et al., 2020). Thus, microbes regulate iron uptake and storage in such a way that restricts the accumulation of ROS in the metabolic process to reduce or prevent damage to the cell from oxidative stress. Therefore, failure to regulate iron concentration could lead to cell damage through the generation of ROS (Pedone et al., 2020).

$$Fe^{+2}+H_{2}O_{2\;}\to OH+OH^{-}+Fe^{+3}\left(\mathrm{Fenton’s reaction}\right)$$ (1)

Ferric uptake regulator (Fur) mediated regulation of SODs has been observed in several bacteria (López-Gomollón et al., 2007). Fur was first identified in *E. coli* as an iron-responsive repressor of iron-transport proteins under iron-replete conditions. Under excessive iron, however, Fur also plays a role in the positive regulation of genes encoding iron-utilizing enzymes (e.g., SodB, SOD) and iron storage proteins (Latifi et al., 2009). Minoshima et al. (2014) demonstrated the role of Fur in enhancing resistance to ROSs by *Thermoplasma volcanium,* a facultative archaeon. Thus Fur encoded by an ORF (TVN0292) [T*. volcanium* Fur protein (TvFur)] resists excessive iron accumulation (Minoshima et al., 2014).

### 5.3 Nitrogen as Nitrosative Stress

Nitrosative stress caused by an increase in reactive nitrogen species (RNS) (Chautrand et al., 2022) is another important stressor during the biodegradation of nitrogen-containing compounds. The main source of RNS is nitric oxide produced by microbial cells during the degradation process. In addition to NO, during denitrification nitrous oxide (N_2_O) is also generated through a reversible reaction and affects microorganisms. Since many dyes contain a nitrogen molecule, complete biological oxidation leads to the production of nitrate, which under anaerobic conditions is converted to N_2_ through denitrification. Therefore, in addition to the generation of ROS during dye degradation, RNS could also be generated and affect microbial activity.

Compared to mesophiles, thermophiles are expected to survive better in the presence of RNS (Martínez-Espinosa, 2020). During wastewater treatment, temperature affects the molecular movement of nitrogenous substances including NO, the sources of RNS, and the dynamics of cell functions. At low temperature, a high concentration of NO could be generated and this affects microbial processes, including denitrification (Rahimi et al., 2020). However, thermophiles have an adaptation to optimally grow at high temperature where a lower amount of RNS is produced. In addition, some thermophilic bacterial strains can detoxify RNS and these properties make them better suited for the treatment of textile industry wastewater (López-García et al., 2015). For example, a thermophilic Campylobacteria strain isolated from Deep-Sea Vent was shown to be capable of reducing N_2_O and NO into nitrogen gas (Fukushi et al., 2020).

### 5.4 Salinity

The interaction of the intracellular and extracellular environment of mesophilic microbial cells is highly affected by high salinity (Aanniz et al., 2015). The effects of salt stress can be reduced by the active transport of ions into the vacuole or out of the cell and by cellular osmolytes regulation. It is believed that under high salinity, the EPS gel becomes thicker, which restricts the diffusion of anions towards the cell (Steele et al., 2014). Since textile industry effluents contain high salinity, thermophiles offer a better potential for the biological treatment of dyes and other pollutants (Mirbolooki et al., 2017).

Steele et al. (2014) demonstrated fluctuating salinity could be managed by extracellular polymeric substances produced by diatoms. The role of EPS in *Cylindrotheca closterium protection* was determined by growing in xanthan gum at different salinities (35, 50, 70, and 90 ppm). The developed biofilm in xanthan (due to EPS matrix) at standard salinity helped cells to maintain function during salinity shock (Steele et al., 2014). In cyanobacterial cells, it is reported that facing salt stress exhibit a high demand for ATP synthesis. Thus, this condition decreases the CO_2_ fixation rate causing the over-reduction of the ferredoxin pool that would result in ROS production leading to oxidative stress (Latifi et al., 2009).

## 6 Conclusion and Future Perspectives

The textile industry uses a variety of dyes for the production of different colored fabrics. However, a significant proportion of the dye ends up into the effluent. Therefore, the release of textile industry wastewater which is loaded with high concentrations of dyes could cause severe environmental pollution. To lower the impact of dyes on the environment, biological treatment of textile industry wastewater is considered essential. However, the process of microbial degradation of dyes leads to the generation of ROS and subjects cells to oxidative stress (OS) that affect microbial activity. Compared to mesophilic microorganisms, thermophiles, because they optimally grow at extreme conditions, develop better adaptation mechanisms to resist the impacts of oxidative stress. Furthermore, a number of thermophilic microbial strains have been shown to be effective in degrading different dyes catalyzed by different thermostable enzymes. This indicates the high potential of thermophiles for application in the treatment of dye containing textile industry wastewater.

One mechanism thermophiles employ to protect themselves against damage by OS is an expression of a range of enzymes involved in the detoxification of ROS. These include superoxide dismutase (SOD), glutathione peroxidase (GPx), and catalase (CAT). Other protective mechanisms include DNA repair system, prevention of the formation of endogenous ROS, activation of the nonenzymatic antioxidant defense system, selective protection of the oxidative damage of certain proteins, and removal and degradation of damaged macromolecules.

Therefore, resistance to oxidative stress and other stressors make thermophiles highly suited for the treatment of different pollutants. Therefore, in addition to their role in the treatment of textile industry effluents, thermophiles have also interesting potential for the treatment of other effluents, such as crude oil and refined petroleum pollutants. However, to date, there is limited study on the use of thermophiles for the treatment of different pollutants. In order to use the full potential of thermophiles for different environmental application, there is a need for detailed understanding of their physiology and the impact of different stress factors on their growth and efficiency. This will require a detailed understanding of regulatory mechanisms and other physiological functions of thermophiles by integrating genomics, transcriptomics, and proteomics approaches.
